# Applications of quantum computing in clinical care

**DOI:** 10.3389/fmed.2025.1573016

**Published:** 2025-04-23

**Authors:** Stevan C. Fairburn, Lara Jehi, Brenton T. Bicknell, Beckley G. Wilkes, Bharat Panuganti

**Affiliations:** ^1^Marnix E. Heersink Institute for Biomedical Innovation, University of Alabama at Birmingham, Birmingham, AL, United States; ^2^UAB Heersink School of Medicine, University of Alabama at Birmingham, Birmingham, AL, United States; ^3^Center for Computational Life Sciences, Cleveland Clinic, Cleveland, OH, United States; ^4^Department of Otolaryngology, Washington University in St. Louis, St. Louis, MO, United States

**Keywords:** quantum computing, clinical care, review, technologic applications in medicine, innovation in medicine

## Abstract

**Introduction:**

This review examines quantum computing (QC) applications in clinical care, emphasizing advancements directly impacting patient outcomes. QC holds transformative potential in medicine, particularly through enhancing diagnostic accuracy, optimizing treatment plans, and enabling real-time decision-making.

**Methods:**

A systematic analysis of 35 studies published between 2015 and 2024 was conducted. The studies were evaluated for their contributions to diagnostic, therapeutic, and decision-support improvements in clinical care enabled by quantum computing technologies.

**Results:**

The analysis revealed QC’s promise in improving diagnostic accuracy in medical imaging, optimizing treatments in oncology, and enhancing real-time clinical decision-making. QC-driven algorithms demonstrated potential to enhance diagnostic accuracy and computational efficiency. These improvements could enable earlier detection of diseases such as Alzheimer’s, cancer, and osteoarthritis, supporting more timely interventions and better prognoses.

**Discussion:**

Despite promising outcomes, current limitations—such as hardware scalability, error mitigation, and ethical considerations—hinder widespread adoption of QC in clinical settings. Overcoming these challenges will require interdisciplinary collaboration and technological innovation. The review underscores QC’s capacity to deliver precise, personalized, and efficient care, advocating for its integration into healthcare workflows to advance precision medicine and improve patient outcomes.

## Introduction

Quantum computing (QC) utilizes the principles of quantum mechanics to novelly process information, rivaling and positioning itself to transcend the capabilities of classical computing. Discussions in the field of medicine are burgeoning around how this technology may enhance research and clinical practice. Recent years have seen a rise in the number of labs, researchers, and clinician-scientists aiming to develop quantum computing with specific applications in the medical field across various specialties and clinical applications.

The computational advantage of quantum computers arises from two key quantum mechanical phenomena: superposition and entanglement. Whereas classical computers utilize bits which operate in binary states (0 or 1), quantum computers employ quantum bits (qubits), which can exist in a superposition of states (0, 1, or both simultaneously). In essence, quantum particles, like electrons or photons, can be in a combination of states, and only upon observation does the system “collapse” into one of these possible states (1). This superposition property enables qubits to represent and process more information than classical bits, which can only exist in a single state at any given time. Entanglement is another fundamental principle, whereby qubits become correlated such that the state of one qubit directly influences the state of another, regardless of the physical distance between them (2). This property allows quantum computers to perform highly coordinated computations with entangled qubits, theoretically increasing the efficiency and power of these complex problem-solving algorithms. Altogether, these properties enable quantum systems to process exponentially more data compared to classical systems. As a result, quantum computing has the potential to problem solve with a different approach that is infeasible for classical computers, solving complex computational problems across various domains, including medicine.

The combination of superposition and entanglement provides quantum computers with a potential advantage over classical systems, particularly in their ability to perform parallel computations. This enables quantum computers to solve complex problems more efficiently, as they can explore multiple possibilities simultaneously, rather than sequentially as classical computers do. In fields such as healthcare, this computational capability is particularly valuable, given vast swaths of underutilized data. For example, quantum computing has the potential to enhance medical imaging techniques, optimize treatment personalization, and improve diagnostic accuracy. Moreover, the ability of quantum systems to handle vast datasets and perform high-dimensional computations positions them as a transformative tool in advancing precision medicine and real-time decision-making in clinical settings. As quantum technology continues to evolve, its applications in healthcare are expected to expand, offering solutions that surpass the limitations of classical computing systems.

Quantum computing, rooted in quantum mechanics, began with Paul Benioff’s 1980 quantum Turing machine model and Richard Feynman and Yuri Manin’s 1982 suggestion that quantum computers could efficiently simulate quantum systems (3–5). By the 2010s, companies like IBM and Google advanced quantum hardware, with IBM offering cloud access to quantum processors (6). A major milestone came in 2019 when Google’s Sycamore processor outperformed classical supercomputers on a specific task (7, 8). Despite progress, challenges like coherence, noise reduction, and scalability persist, and these challenges are highly accentuated in implementation of QC within a healthcare environment that has a low tolerance for risk and high value on patient safety (9, 10). However, promising developments, such as MIT’s demonstration of quantum entanglement in a 4x4 superconducting qubit array, hint at transformative potential for drug discovery and healthcare, and point toward QC as a means of solving some of healthcare’s most pressing challenges (11).

Quantum computing is a rapidly advancing field with applications in physics, cryptography, material science, logistics, and AI, tackling problems like supply chain optimization, machine learning enhancement, and molecular simulations for drug discovery. While its potential in healthcare spans across medical imaging, personalized treatment, genomics, and real-time decision-making, research in clinical medicine remains limited. As the technology progresses, understanding its direct applications in clinical care becomes increasingly crucial.

This paper aims to provide a comprehensive review of the current and potential applications of quantum computing in clinical care, addressing a gap in the literature that often overlooks its direct clinical relevance. This review seeks to consolidate existing research on quantum computing applications across various medical fields and specialties. Additionally, the review will explore the technological challenges that need to be overcome to integrate quantum computing into clinical practice, offering valuable insights for healthcare professionals, researchers, and policymakers.

## Methods

### Research question

This review aims to explore the applications of quantum computing in clinical care. This review was formulated to ensure comprehensiveness while focusing on identifying practical applications of quantum computing in improving patient care and clinical outcomes, particularly in medical imaging, diagnostic precision, treatment personalization, and real-time decision-making in healthcare.

### Search strategy

The search strategy for this review followed a methodological framework, leveraging a comprehensive search across three major databases (PubMed, Scopus, Web of Science) for literature published from January 2015 to January 2024. A combination of keywords and MeSH terms were used, including “quantum computing,” “clinical medicine,” “clinical care,” “quantum computers,” “medical imaging,” “diagnostic precision,” “treatment personalization,” “quantum machine learning,” “quantum encryption,” “bioinformatics,” “surgical planning,” “simulation,” and “real-time decision-making.”

### Study selection

Article types for consideration included peer-reviewed original research articles, review/overview articles, and theoretical papers that propose or implement novel applications of quantum computing in clinical care. Although review and opinion articles are generally omitted from reviews, we included these articles due to the rapidly expanding, yet still small, body of original research to provide the reader with a more comprehensive overview of the topic. To be included in this review, studies needed to propose or demonstrate practical applications of QC that directly impact clinical care, be published within the specified timeframe, and be documented in English in peer-reviewed journals. We excluded articles that lacked direct clinical relevance, those focusing on theoretical aspects of QC without clear medical applications, duplicates, and non-English language publications. Clinical relevance was defined as a quantum computing application that addressed a first order impact in the way that care could be delivered in a clinical medicine setting.

Titles and abstracts of retrieved articles were independently screened by two reviewers. These reviewers assessed relevance based on inclusion criteria: focus on quantum computing applications in clinical care, publication in English, within the specified date range, and sourced from the identified databases. Exclusion criteria included lack of direct clinical relevance, publication outside the date range, non-English language, lack of focus on quantum computing, or duplicates. Discrepancies were resolved through discussion or consultation with a third reviewer. Articles passing the initial screening were subjected to full-text reviews by the same reviewers. This stage assessed the relevance and quality based on detailed inclusion and exclusion criteria. Studies that did not meet the inclusion criteria upon closer inspection were excluded during this phase. Articles regarding drug discovery were largely excluded given the second order impact on clinical care. However, articles with a primary application in clinical settings within the realm of drug discovery were included as they were deemed to have a first order impact on clinical care.

### Data extraction

We developed a standardized data extraction form to maintain consistency while capturing relevant information from each selected article. The form was designed to collect comprehensive details including bibliographic information (authors, title, publication year, journal), study design, objectives, discussed applications of quantum computing, clinical relevance, methodologies, results, and conclusions.

Each article was meticulously reviewed, and data were extracted independently by two reviewers using the standardized form. This dual-review approach enhanced the accuracy of the data collected and helped minimize bias. Key information extracted included details on the specific applications of quantum computing in clinical care, integration with other technologies like AI and bioinformatics, and any reported outcomes or future implications of the studies. The quality of each study was evaluated against predefined criteria focused on the clarity of objectives, methodological rigor, and the relevance of findings to clinical care. Studies were categorized based on their level of evidence, and potential biases were identified and documented, ensuring that only high-quality data influenced the review’s conclusions. The final set of articles was compiled after achieving a consensus between the reviewers. Articles selected were deemed to have significant relevance to the application of quantum computing in clinical care.

### Data synthesis

The data extracted were synthesized to provide a comprehensive overview of the current research landscape regarding quantum computing applications in clinical care. Thematic analysis that simultaneously noted both application of quantum computing and clinical relevance was used to identify common themes across the studies, including areas such as medical imaging, diagnostic precision, drug discovery, genomic analysis, surgical planning, and treatment personalization.

Key themes were identified, and gaps in the literature were noted. The synthesis also included recommendations for future research directions, highlighting areas where quantum computing may have the most significant impact on clinical care.

## Results

### Overview of included studies

**FIGURE 1:**
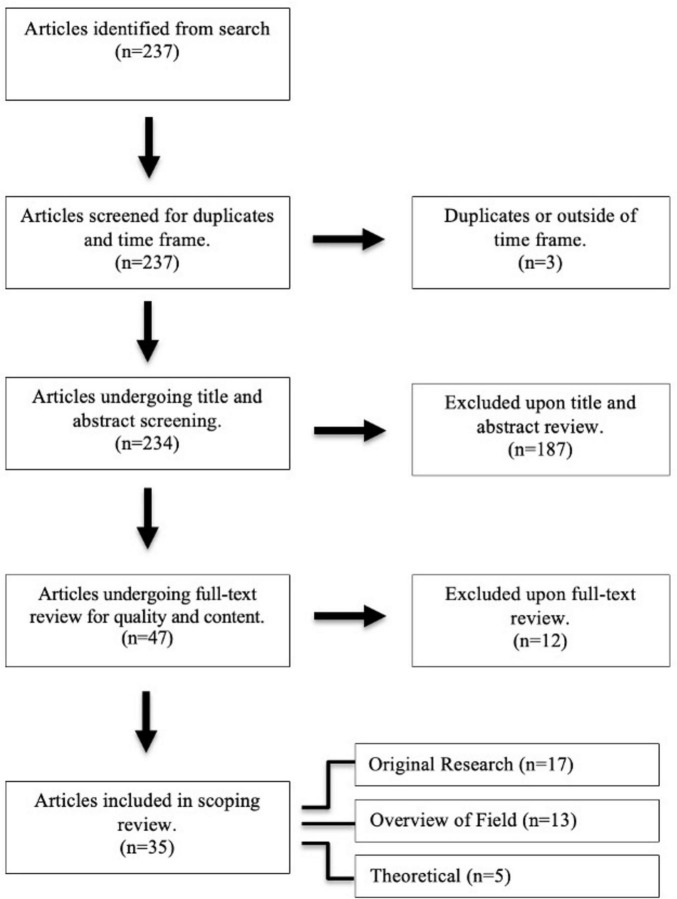
PRISMA flow diagram for article selection and review process. This diagram outlines the selection process for this scoping review on quantum computing in clinical care. A total of 237 articles were identified from database searches. After removing duplicates, 234 unique articles were screened by title and abstract. Full-text review for content and quality were conducted on the remaining 47 articles. The final selection included 35 articles, which were thoroughly reviewed for relevance and quality.

From the initial 237 publications identified, 35 met the inclusion criteria for this review. Figure 1 outlines the selection process, including the screening and eligibility stages. It also provides a breakdown of the 35 sources based on article type.

### Clinical applications of quantum computing

The studies reviewed demonstrated a broad range of applications of quantum computing being investigated in clinical care (Table 1). The most frequently discussed topics were medical imaging (54.3%) and clinical decision-making (54.3%). Cancer-related applications were also prominent, with 48.6% of studies focusing on quantum computing’s role in cancer detection, treatment planning, and understanding cancer mechanisms. Personalized medicine (37.1%) and predictive modeling (22.9%) were additional key topics where quantum computing was identified as having potential to tailor treatments and forecast disease outcomes based on patient-specific data. Other areas of investigation included microbiology, genomics, and cybersecurity, reflecting the diverse and growing interest in quantum computing’s utility across multiple clinical healthcare applications (Table 1).

**TABLE 1:** Articles discussing quantum computing clinical applications categorized by clinical application.

Clinical application	No. (%) of articles
**Core clinical applications**	
Medical imaging	19 (54.3)
Clinical decision making	19 (54.3)
**Disease-specific applications**	
Biopsy	1 (2.9)
Cancer	17 (48.6)
Microbiology	5 (14.3)
**Personalized & predictive healthcare**	
Personalized medicine	13 (37.1)
Predictive modeling	8 (22.9)
**Pharmacology & genomics**	
Pharmacology	8 (22.9)
Genomics & genome editing	5 (14.3)
**Emerging and supporting technologies**	
Cybersecurity	4 (11.4)

No, number.

### Investigations across specialties

Quantum computing research spans a variety of medical specialties (Table 2), with the most significant focus on radiology (51.4%) and oncology (48.6%). In radiology, quantum computing is primarily being explored for its potential to improve imaging techniques and diagnostic accuracy, while in oncology, it is being investigated for cancer detection, treatment planning, and personalized care. Neurosurgery (20.0%) has also attracted attention, particularly in enhancing precision during surgical procedures. Other specialties, such as genomics (14.3%) and infectious diseases (14.3%), are also exploring quantum computing’s potential for large-scale data analysis and disease modeling. Additional fields with emerging research interest include cardiology, general surgery, urology, and neurology, each contributing smaller but notable efforts to examine the clinical applications of quantum computing (Table 2).

**TABLE 2:** Articles discussing quantum computing clinical applications categorized by medical specialty.

Specialty	No. (%) of articles
**Medical specialties**	
Cardiology	3 (8.6%)
Dermatology	1 (2.9%)
Endocrinology	1 (2.9%)
Genomics	5 (14.3)
Infectious disease	5 (14.3)
Neurology	2 (5.7%)
Oncology	17 (48.6%)
Pathology	2 (5.7%)
Radiology	18 (51.4%)
Urology	2 (5.7%)
**Surgical specialties**	
General surgery	3 (8.6%)
Neurosurgery	7 (20.0%)
Otolaryngology	1 (2.9%)

No, number.

### Temporal trends of research in quantum computing in medicine

**FIGURE 2:**
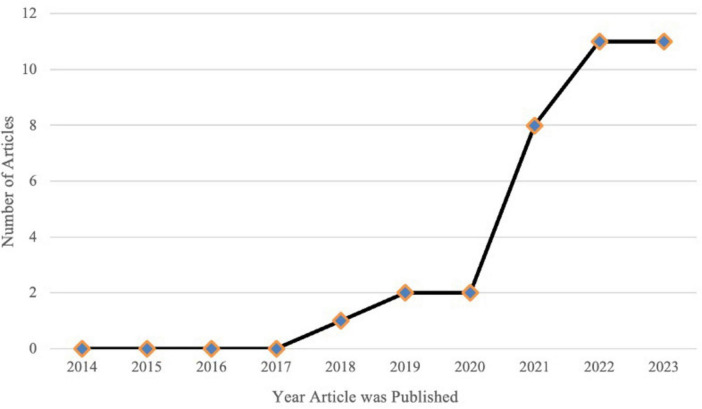
Temporal analysis of included articles. The number of publications related to the clinical applications of quantum computing has increased significantly over time. Linear regression analysis shows a statistically significant upward trend, with an estimated 1.33 additional publications per year (*B* = 1.327, 95% CI, 0.685 to 1.969; *p* = 0.001), indicating growing research interest and expansion in this field.

As shown in Figure 2, there has been a significant upward trend in the number of publications related to quantum computing in clinical care over time. Linear regression analysis demonstrated that 74% of the variance in the number of publications was explained by the year (R^2^ = 0.740). The number of publications increased by an estimated 1.33 per year (OR = 1.327, 95% CI, 0.685 to 1.969; *p* < 0.01). The model was statistically significant (*F* = 22.725, *p* < 0.01).

### Study limitations

This systematic review has several inherent limitations. First, the search strategy was limited to PubMed, Scopus, and Web of Science; therefore, relevant studies published in other databases, conference proceedings, or gray literature may have been missed, impacting comprehensiveness. Furthermore, most reviewed studies remain theoretical, simulation-based, or utilize synthetic datasets, significantly limiting the immediate translation and clinical applicability of QC findings to real-world patient care. The considerable heterogeneity across clinical outcomes, algorithms evaluated, and medical specialties reviewed complicates synthesis and generalizability of results. Additionally, due to QC’s novelty, there is potential for positive-outcome bias, as studies reporting less favorable outcomes may be underrepresented. Another substantial challenge involves the lack of standardized benchmarks to quantitatively compare quantum computing methods with classical counterparts consistently across clinical contexts. Future systematic reviews should employ expanded search strategies, advocate for standardized benchmarks, and emphasize longitudinal studies validating QC approaches against clinical outcomes. Despite these limitations, this review provides important insights into QC’s promising future role in clinical medicine.

## Discussion

Quantum computing is poised to revolutionize clinical care by addressing computational bottlenecks and enhancing the precision, scalability, and adaptability of medical workflows. The studies reviewed exemplify diverse and innovative applications of quantum technologies in healthcare, spanning diagnostics, treatment planning, imaging, genomics, and pharmacology. Together, they underscore the potential of quantum computing to transform the practice of medicine, while also highlighting that quantum computing remains largely in the research phase, with significant milestones to achieve before widespread clinical adoption.

### Enhanced diagnostic accuracy and efficiency

Several studies demonstrated the utility of quantum-enhanced models in improving diagnostic workflows. For instance, the hybrid classical-quantum neural networks (HCQNN) for Alzheimer’s detection, trained on a balanced dataset of 6400 MRI scans labeled as demented and non-demented, achieved over 97% classification accuracy validated using quantum simulators. The quantum layer’s dimensionality reduction, combined with a ResNet34-based classical feature extractor, enabled precise decision boundaries, outperforming the classical-only model’s 92% accuracy with a significant improvement in computational efficiency (12). Similarly, a cardiomegaly detection model integrated pre-trained DenseNet-121 with quantum circuits, using a balanced subset of 2436 posteroanterior chest X-rays from the CheXpert repository. It achieved a 10% improvement in classification accuracy over classical models while leveraging trustworthy Grad-CAM++ visualizations to address imbalanced datasets and enhance clinical interpretability (13).

Quantum computing’s potential in dermatological applications was highlighted through its use in skin lesion analysis. Using the ISIC 2019 dataset, a quantum-enhanced Inception-ResNet-V1 model, with quantized convolutional layers and SVM classifiers, achieved 98% accuracy in multi-class skin lesion classification, outperforming classical CNN models which ranged between 81 and 97%, highlighting quantum computing’s superior accuracy and computational efficiency. This significantly outperformed traditional visual assessments by clinicians, which are often subjective and influenced by variability in experience and conditions. Quantum computing enhanced the diagnostic process by leveraging quantum parallelism for efficient feature extraction, enabling the identification of subtle patterns in large datasets and reducing computational overhead. This advancement underscores quantum models’ ability to improve diagnostic accuracy and preventive medicine by identifying potentially malignant lesions earlier and more reliably (14).

Quantum-based approaches also show promise in assessing disease progression in osteoarthritis using knee imaging datasets. This study quantitatively compared quantum-to-classical transfer learning methods with hybrid quantum convolutional neural networks (HQCNN) alone for knee osteoarthritis classification, demonstrating a measurable improvement of 1.08%, achieving a remarkable overall accuracy of 98.36%. By leveraging sparse quantum feature selection, these models achieved superior classification accuracy compared to traditional methods, enabling earlier detection of degenerative changes (15).

In endocrine oncology, quantum computing demonstrated significant potential by enhancing imaging precision and identifying subtle differences in endocrine tumor pathology. Quantum-enhanced imaging techniques have paved the way for improving early detection and surgical planning (16). Quantum computing also shows promise in aiding clinical decision-making in oncology. Quantum-enhanced algorithms demonstrated the ability to integrate multiple patient data types, enabling personalized treatment recommendations and improved decision-making processes in complex cancer cases (17).

The contribution of overview articles underscores the importance of quantum computing in diagnostics. For example, one overview highlighted the potential of quantum-enhanced imaging for early-stage disease detection, detailing case studies where quantum models accelerated imaging processes and reduced diagnostic delays (18). Another overview emphasized the utility of quantum-enhanced machine learning in diagnosing COVID-19 pulmonary complications, demonstrating rapid identification and triage capabilities during pandemics (19).

Theoretical perspectives added depth to these findings by proposing frameworks such as quantum-enhanced image processing for histopathology slides, allowing rapid identification of cellular abnormalities in diseases like cancer (20). These advancements in diagnostic capabilities hold promise for improving outcomes in preventive medicine, where early detection plays a critical role in reducing disease burden and improving survival rates.

### Advancing radiomics and imaging workflows

Quantum computing’s application in radiomics and imaging emerged as a significant theme across the studies. Quantum error mitigation techniques (strategies to reduce computational errors inherent in quantum systems) applied to PET radiomic cancer characterization allowed quantum models to achieve accuracies comparable to classical methods. They demonstrated that quantum methods achieved higher accuracy (70%) compared to classical methods (69%) with 16 features. Furthermore, employing quantum hardware with error mitigation resulted in a substantial improvement in balanced accuracy (from 69.94% without mitigation to 75.66% with mitigation), closely matching noiseless simulation results, thus highlighting quantum computing’s practical advantage (21). Similarly, emission tomography reconstruction using hybrid adiabatic quantum computing (a method that gradually transitions quantum systems from a simple starting point to a more complex solution state, often used to solve optimization problems) provided critical insights into the scalability of quantum methods, particularly for low-complexity scenarios, while identifying the need for improved quantum hardware to handle high-resolution imaging demands (22).

An overview article provided additional insights into radiation oncology, focusing on quantum-enhanced segmentation tools for tumor delineation. These tools were shown to improve radiotherapy planning by tailoring radiation doses to individual patients, emphasizing the need for integration into clinical workflows (23). Theoretical studies supported this by discussing potential applications of quantum simulations in adaptive radiotherapy to refine dose distributions dynamically (24). More granular examples include QNNs used to identify complex tumor geometries with enhanced precision and quantum-based adaptive imaging tools to improve real-time adjustments during treatment planning (25).

Neurosurgical planning leverages quantum computing to address computationally intensive tasks like real-time image registration and intraoperative updates. For example, quantum algorithms enhance physics-based non-rigid registration (PBNRR) methods used to align pre- and intraoperative MRI scans by significantly reducing the computational load and improving the precision of brain deformation tracking during surgery. These quantum-enhanced approaches improve registration accuracy, reducing errors by up to 2.5 mm compared to classical methods, and meet the stringent real-time requirement of completing computations in under 4 min (26). This improved accuracy and speed enable surgeons to better localize critical brain structures and make intraoperative adjustments, enhancing the safety and effectiveness of neurosurgical procedures (27).

### Precision in oncology

Quantum computing holds tremendous potential for oncology, as evidenced by multiple studies. Quantum neural networks (QNNs) for differentiating large brain metastases from high-grade gliomas leveraged quantum annealing (a process that uses quantum fluctuations to search for optimal solutions to specific computational problems, such as feature selection) to enhance feature selection. The quantum method achieved balanced accuracy (0.74) comparable to classical dense neural networks (0.73) and extreme gradient boosting (0.72), demonstrating similar or slightly superior generalization on the test dataset (25). The explainable nature of this approach increases its clinical applicability, as clinicians require insight into why models make specific predictions.

Quantum-inspired recurrent neural networks (QRNNs) accurately predicted geometric changes in head and neck cancer during radiotherapy, a critical capability for adapting treatment plans to tumor dynamics (28). Additionally, hybrid quantum-classical models for drug response prediction demonstrated a 15% improvement in the accuracy of predicting IC50, which represents the concentration of a drug required to inhibit a biological process by 50%, highlighting their potential for advancing precision oncology by tailoring therapies to individual patients (29).

Overviews further emphasize the role of quantum computing in oncology. For example, one overview highlighted the use of quantum optimization algorithms for balancing radiation dose and toxicity in radiotherapy, showing how these models can improve treatment precision and reduce side effects (30). Another overview focused on QML for analyzing radiomic and genomic datasets, demonstrating its superior capability in classifying cancer subtypes and predicting treatment outcomes (31). Theoretical papers complemented these findings by proposing methodologies for quantum-assisted tumor modeling to predict therapy resistance (32).

Quantum computing has shown promise in tuberculosis (TB) treatment by enabling precise modeling of pathogen dynamics and optimizing therapeutic strategies to counter drug resistance. For example, one approach uses counterdiabatic quantum control to guide genotype distributions in evolving TB populations. This technique allows for targeted evolution under varying drug regimens, accelerating the transition to desired resistance profiles and informing adjustments to antimicrobial therapies, which is critical for managing multidrug-resistant and extensively drug-resistant TB (33). Additionally, the integration of quantum computing into diagnostic tools, such as molecular imaging and rapid genome analysis, enhances the ability to detect TB earlier and more accurately, supporting improved treatment outcomes and guiding the development of more effective prophylactic strategies (34).

In spine care, quantum computing enhances predictive analytics by processing complex datasets that traditional methods cannot handle. Quantum-assisted models integrated with AI improve preoperative planning by predicting patient-specific risks and complications with greater precision, enabling more personalized and effective surgical care (35). In conjunction with quantum-assisted approaches for studying versatile molecular pathways like Wnt/β-Catenin in cancer, these advancements provide broader insights into disease management and personalized therapy (36).

### Genomics and molecular modeling

Quantum computing demonstrated its ability to accelerate genomic and molecular analyses, which are foundational to precision medicine. The quantum gate algorithm accelerated DNA sequence alignment while maintaining accuracy comparable to BLAST, a widely used classical method (37). This improvement is clinically relevant as it enables faster and more accurate processing of genomic data, essential for personalized medicine applications such as identifying genetic mutations linked to hereditary diseases or tailoring targeted therapies based on an individual’s genetic profile.

Quantum-assisted molecular modeling for SARS-CoV-2 protein docking used the Quantum Approximate Optimization Algorithm (QAOA, a hybrid quantum-classical algorithm designed to find optimal solutions for problems such as molecular docking by iteratively refining a solution) to identify high-affinity binding sites efficiently, paving the way for the rapid development of therapeutic targets during pandemics (38). Similarly, quantum neural networks applied to fMRI pain decoding improved feature extraction and regression accuracy, demonstrating robust applications in neurological research and care (39).

Overview articles further explored quantum computing’s potential in drug delivery systems, particularly in designing targeted mechanisms for chronic diseases and oncology. Quantum simulations were proposed to predict molecular interactions with unprecedented accuracy, facilitating personalized treatment regimens (40). Theoretical models outlined quantum-enhanced multi-modal analyses, integrating genomics, proteomics, and transcriptomics to derive holistic insights into disease mechanisms (41).

### Tackling computational bottlenecks in medical workflows

A recurring theme across the studies was the use of quantum computing to overcome computational bottlenecks in healthcare. The Quantum-Behaved Sparse Dictionary Learning (QMVO-SCDL) model for pain decoding utilized quantum behaviors like superposition to optimize feature selection, allowing models to evaluate multiple potential solutions at once, outperforming traditional regression methods in predictive reliability (39). The integration of quantum circuits into diagnostic and therapeutic models streamlined workflows, enabling faster, more precise decision-making. For example, the quantum-enhanced classification models for cardiomegaly and Alzheimer’s demonstrated the practical potential of integrating quantum layers into existing AI architectures, making diagnostic tools more scalable and efficient (12, 13).

Quantum metric learning classifiers for breast cancer diagnosis demonstrated improvements in generalization performance by addressing feature dimensionality, which is critical in minimizing overfitting and enhancing clinical applications of predictive models. This study quantitatively demonstrated that quantum metric learning classifiers, when properly optimized, achieved F1 scores as high as 0.9722 and significantly lower test costs compared to classical methods. Notably, quantum methods showed enhanced generalization performance and computational efficiency, especially when combined with dimensionality reduction techniques, thus highlighting the potential clinical benefit of quantum computing (42).

Quantum data classification in clinical medicine showed advancements in addressing noise and variability across oncology and cardiology datasets. These applications optimized diagnostic reliability and predictive accuracy, particularly in high-stakes medical decisions (43).

### Cybersecurity, data integration, and resource allocation

As quantum computing continues to advance, its implications for cybersecurity and data integration become increasingly critical. Review findings highlighted the use of quantum encryption techniques, such as quantum key distribution (QKD), to secure sensitive patient data against quantum-enabled cyberattacks (44). Theoretical proposals suggested creating Quantum Universal Exchange Languages (QUEL) to enhance interoperability between quantum and classical systems, facilitating seamless data sharing in multi-center clinical trials (32). Additionally, quantum-assisted resource allocation frameworks demonstrated potential for improving hospital workflows, such as dynamically managing operating room schedules and optimizing patient triage processes in real time (45).

### Real-world implications for clinical care

While quantum computing remains in the research stage, these studies demonstrate its potential to redefine clinical workflows. In diagnostic imaging, quantum models could significantly reduce processing times, enabling real-time decision-making in critical care settings (18, 19). In oncology, quantum-enhanced algorithms offer the promise of more personalized treatment plans, optimizing dosages, and reducing side effects (30, 31). Genomics applications, such as DNA sequencing and molecular docking, could accelerate drug discovery and improve the precision of targeted therapies (37, 38).

Insights from overviews further contextualize these implications, highlighting the commercial applications of quantum computing in healthcare logistics, such as optimizing patient scheduling and resource allocation, which can enhance operational efficiency (46). Ethical considerations are also critical, as quantum computing introduces new challenges regarding transparency, accountability, and equitable access to these transformative technologies. Preventive medicine is another key area, with quantum-enhanced methods enabling earlier disease detection and intervention strategies to improve population health outcomes.

The transformative potential of quantum computing in clinical workflows parallels the historical role medical physicists played in revolutionizing radiation oncology. Prior to their integration, radiation therapy relied heavily on empirical methods with limited precision, significantly constraining treatment efficacy and patient safety. The introduction of medical physicists brought advanced computational dosimetry, precise radiation modeling, and rigorous quality assurance protocols, allowing for individualized treatment plans with unprecedented accuracy. This shift dramatically improved therapeutic outcomes, reduced toxicity, and established radiotherapy as a cornerstone of modern cancer care. Similarly, quantum computing experts, working closely with clinicians, could fundamentally reshape healthcare workflows across medical disciplines. By providing clinicians with computational tools capable of efficiently analyzing complex datasets, quantum computing could enhance diagnostic precision, optimize personalized treatments, and support real-time clinical decision-making, ultimately resulting in safer, more effective, and individualized patient care.

Consider the scenario of neurosurgical resection of a glioblastoma intimately adjacent to critical motor pathways. Currently, neurosurgeons depend on preoperative imaging, intraoperative navigation, and electrophysiological monitoring; however, subtle shifts in brain anatomy during surgery pose substantial challenges, potentially compromising surgical outcomes. Quantum computing could offer innovative solutions. In close collaboration with quantum physicists, neurosurgeons could leverage quantum-enhanced algorithms to rapidly integrate preoperative MRI, diffusion tensor imaging (DTI), and functional MRI (fMRI) data with real-time intraoperative imaging. Harnessing quantum parallel-processing capabilities, these algorithms would instantaneously update precise anatomical maps, accurately capturing intraoperative changes within seconds—far exceeding the speed and precision achievable through classical computing methods. This quantum-driven precision significantly reduces risks to adjacent healthy tissue, enabling surgeons to perform safer, more complete tumor resections, thereby improving functional preservation, shortening recovery times, and enhancing patients’ postoperative quality of life. Such interdisciplinary partnerships exemplify quantum computing’s promising role in revolutionizing clinical outcomes.

### Future directions and implications

The research reviewed highlights quantum computing’s ability to address critical challenges in clinical care, but it also reveals areas for further exploration. While the advancements discussed are promising, quantum computing remains at an early stage of development, with its implementation in clinical workflows still years away. Challenges such as hardware noise, error rates, and the limited scalability of current quantum systems must be addressed before these technologies can achieve widespread adoption.

Future efforts should focus on refining quantum algorithms to operate reliably under practical constraints. For example, scalable approaches to DNA sequence alignment and emission tomography reconstruction demonstrate the need for robust methodologies that can translate well from research environments to real-world clinical applications (22, 37). Furthermore, integrating error mitigation techniques is essential to ensuring the reliability of quantum models, particularly in high-stakes medical decision-making (21).

Despite these challenges, the trajectory of quantum computing research signals transformative possibilities. For instance, advancements in quantum machine learning could enable faster, more precise diagnostics, while quantum-enhanced molecular modeling has the potential to accelerate clinically meaningful drug discovery and improve treatment personalization. The interdisciplinary nature of these developments—bridging quantum computing, artificial intelligence, and clinical science—underscores the importance of collaborative efforts to maximize their impact.

In conclusion, while quantum computing is not yet ready for direct integration into clinical practice, its ongoing evolution holds tremendous potential for the future of medicine. By addressing current limitations and continuing to build upon foundational research, the field can move closer to creating practical, impactful tools that redefine diagnostics, treatment planning, and biomedical research.

## Data Availability

The original contributions presented in this study are included in this article/supplementary material, further inquiries can be directed to the corresponding author.
